# Crystal structure of tetra­phenyl phosphate tetra­kis­[dimethyl (2,2,2-tri­chloro­acet­yl)phos­pho­ramidato]lutetium(III), PPh_4_[Lu*L*_4_]

**DOI:** 10.1107/S205698902400210X

**Published:** 2024-03-12

**Authors:** Mariia B. Struhatska, Vladimir A. Ovchynnikov, Nataliia S. Kariaka, Paula Gawryszewska, Volodymyr M. Amirkhanov

**Affiliations:** a Department of Chemistry, Taras Shevchenko National University of Kyiv, Volodymyrska Street 64, Kyiv 01601, Ukraine; b Faculty of Chemistry, University of Wroclaw, 14 F. Joliot-Curie Street, 50-383, Wroclaw, Poland; Universidad de Los Andes Mérida, Venezuela

**Keywords:** crystal structure, lanthanide, carbacyl­amido­phosphate CAPh, rare-earth metals, coordination compound, tetra­kis complex, chelate ligand, lutetium, tetra­phenyl­phospho­nium cation

## Abstract

A lutetium(III) complex based on the anion of the ligand dimethyl (2,2,2-tri­chloro­acet­yl)phospho­ramidate (H*L*) and tetra­phenylphosphonium, of composition PPh_4_[Lu*L*_4_] (*L* = CAPh = carbacyl­amido­phosphate), or (C_24_H_20_)[Lu(C_4_H_6_Cl_3_NO_4_P)_4_], has been synthesized and structurally characterized. The X-ray diffraction study of the compound revealed that the lutetium ion is surrounded by four bis-chelating CAPh ligands, forming the complex anion [Lu*L*_4_]^−^ with a coordination number of 8[O] for Lu^III^, while PPh_4_^+^ serves as a counter-ion. The coordination geometry around the Lu^3+^ ion was determined to be a nearly perfect triangular dodeca­hedron. The complex crystallizes in the monoclinic crystal system, space group *P*2_1_/*c*, with four mol­ecules in the unit cell. Weak hydrogen bonds O⋯HC(Ph), Cl⋯HC(Ph) and N⋯HC(Ph) are formed between the cations and anions. For a comparative study, H*L*-based structures were retrieved from the Cambridge Structural Database (CSD) and their geometries and conformations are discussed. A Hirshfeld surface analysis was also performed.

## Abstract 2

The crystal structure of the anionic lutetium(III) tetra­kis-CAPh complex (CAPh = carbacyl­amido­phosphate) with tetra­phenyl phosphate as the cation, PPh_4_[Lu*L*_4_], is presented and discussed.

## 1. Chemical context

Luminescent coordination compounds of lanthanides have attracted significant attention due to their diverse potential applications in lighting technology, including fluorescent lamps, LEDs, displays, telecommunications, lasers, sensors, luminescent probes for biological applications, for solar energy conversion and photocatalysis (Binnemans, 2009 ▸). Some of the extensively investigated ligands used for binding lanthanide(III) ions include β-diketones and compounds structurally akin to them (Nehra *et al.*, 2022 ▸; Duan *et al.*, 2022 ▸; Magennis *et al.*, 1999 ▸). Within this category, a noteworthy subset comprises ligands known as carbacyl­amido­phosphates (CAPhs), which incorporate a functional unit C(O)NHP(O), and enable bidentate chelation upon coordination. The inclusion of the phosphoryl group in CAPhs imparts a strong affinity for lanthanides (Amirkhanov *et al.*, 2014 ▸). In this work, we intended to design a new lutetium(III) CAPh-based tetra­kis complex with a bulk cation in order to obtain it in a crystalline form and investigate a quite rare example of a lutetium complex structure. From this idea, the compound PPh_4_[Lu*L*_4_] was synthesized in high yield *via* reaction between lutetium nitrate, tetra­phenyl­phospho­nium bromide and the sodium salt of the ligand Na*L*. 
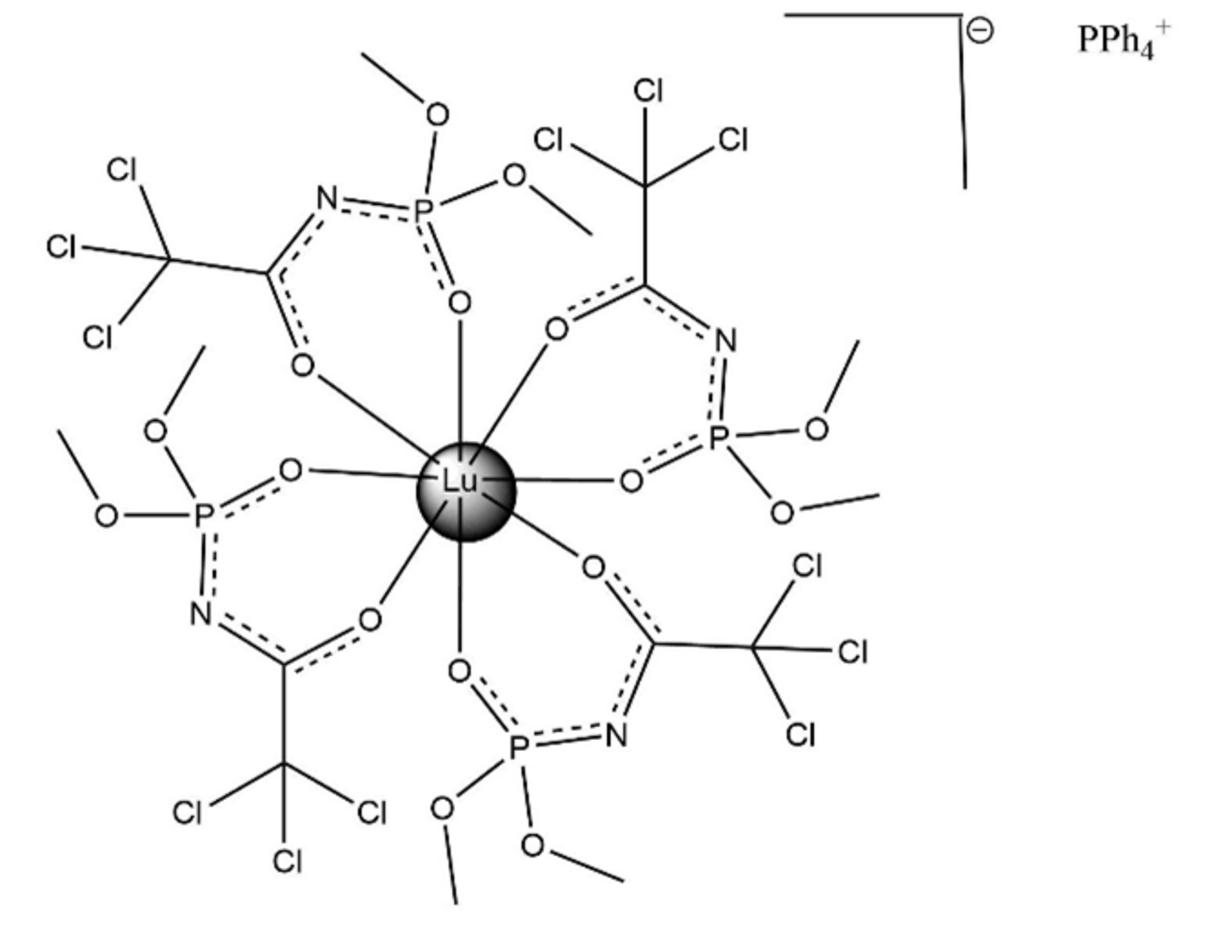


## 2. Structural commentary

**Figure 1:**
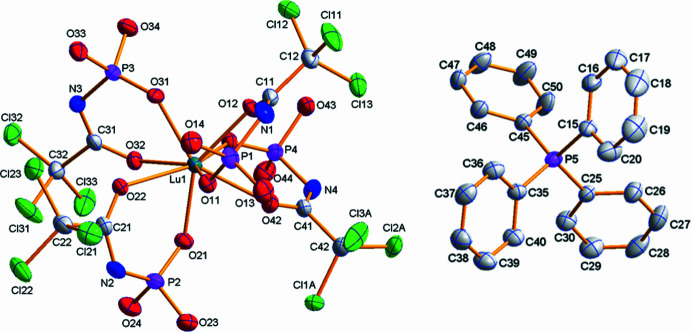
The mol­ecular structure of the title compound with displacement ellipsoids drawn at the 50% probability level. Alkyl groups of the ligand and hydrogen atoms are omitted for clarity.

**Figure 2:**
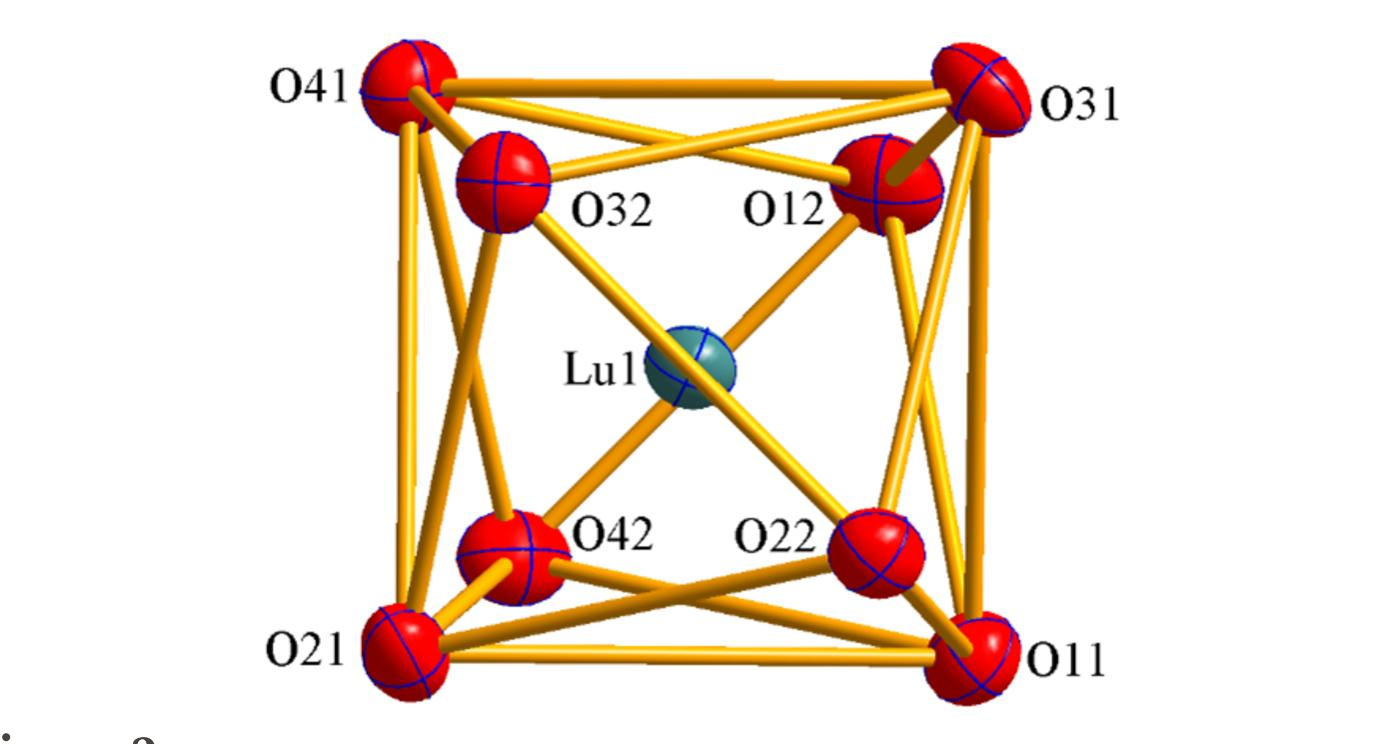
Coordination environment of the lutetium(III) ion.

The title compound (C_24_H_20_P)[Lu(C_4_H_6_Cl_3_NO_4_P)_4_] crystallizes in the monoclinic system in space group *P*2_1_/*c* with four mol­ecules in the unit cell. All four ligands are coordinated in a bidentate chelate manner through the oxygen atoms of the carbonyl and phosphoryl groups. The complex comprises the [Lu*L*_4_]^−^ anion and the PPh_4_^+^ counter-ion, which are inter­connected by hydrogen bonds (Table 1 ▸) and weak inter­molecular inter­actions. The mol­ecular structure of the complex is shown in Fig. 1 ▸ and the coordination polyhedron in Fig. 2 ▸. The coordination polyhedron of the Lu^3+^ ion was determined to be a nearly perfect triangular dodeca­hedron formed by the eight O atoms of the bidentate CAPh ligands. The calculation was carried out using *SHAPE 2.1* (Llunell *et al.*, 2013 ▸).

The average Lu—O bond length in PPh_4_[Lu*L*_4_] is 2.3116 Å, which is longer than in {Lu_2_ *L*_6_·μ-(γ, γ′-dipy)} (2.2403 Å; Trush *et al.*, 2001 ▸). The Lu—O(C) bond lengths [2.348 (3)–2.411 (2) Å] are all longer than the Lu—O(P) bonds [2.236 (3)–2.267 (3) Å], which is explained by higher affinity of the phosphoryl group towards the metal ion. Deprotonation of the ligands leads to an increase of the π-conjugation in the chelating fragments and results in changes in the bond lengths. The C—O and P—O bond lengths are shorter than in the binuclear Lu^III^ complex and are in the ranges 1.233 (4)–1.245 (5) Å and 1.483 (3)–1.489 (3) Å, respectively, with corresponding average values of 1.2399 and 1.486 Å. In contrast, in {Lu_2_L_6_·μ-(γ, γ′-dipy)} (Trush *et al.*, 2001 ▸) the C—O bond lengths lie within 1.237–1.258 Å (average 1.247 Å) and the P—O bond lengths lie between 1.492 and 1.509 Å (average 1.501 Å). The corresponding bond lengths in the neutral ligand H*L* are 1.202 (2) and 1.459 (2) Å (Amirkhanov *et al.*, 1995 ▸ *)*. The C—O and P—O bonds in the complex are longer than those in the neutral ligand (H*L*), indicating greater C—O and P—O double-bond character in H*L* than in the complex. The C—N and P—N bonds in PPh_4_[Lu*L*_4_], with lengths in the ranges 1.295 (6)–1.315 (5) and 1.613 (3)–1.624 (4) Å, respectively, are shorter compared to those in the free ligand, in which the reported C—N bond length is 1.347 (2) Å and P—N is 1.676 (1) Å. The C—N bond lengths in the binuclear lutetium complex are proportional to those in the *tetra­kis*- and lie between 1.297 and 1.314 Å while the P—N distances are shorter (1.602–1.621 Å).

## 3. Supra­molecular features

**Figure 3:**
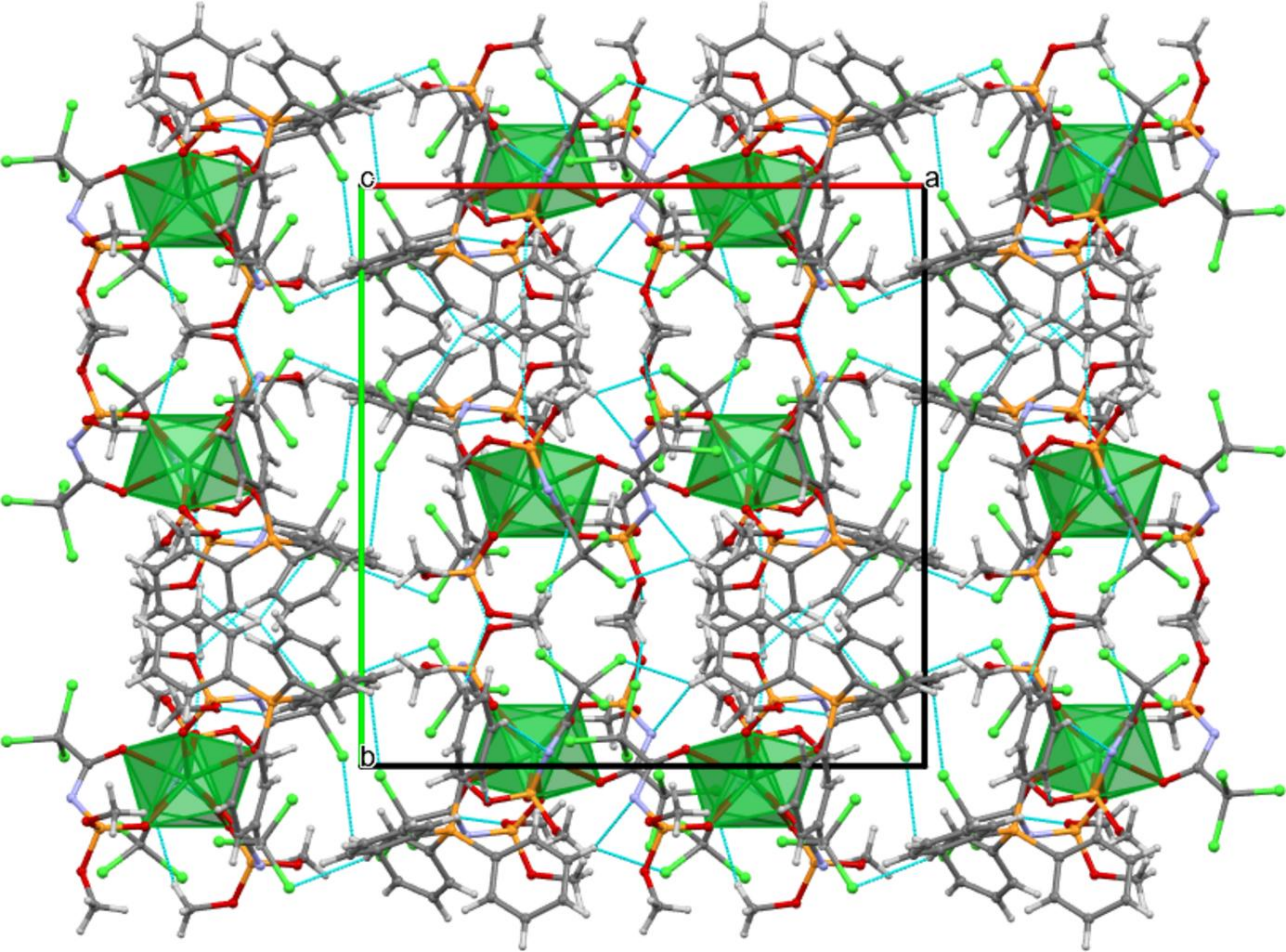
The crystal packing of the title compound viewed down the *c*-axis. Hydrogen bonds are shown as dashed cyano lines.

The crystal packing of the title compound viewed down the *c*-axis is shown in Fig. 3 ▸. The Lu^III^ polyhedra are isolated and do not share edges or vertices.

**Figure 4:**
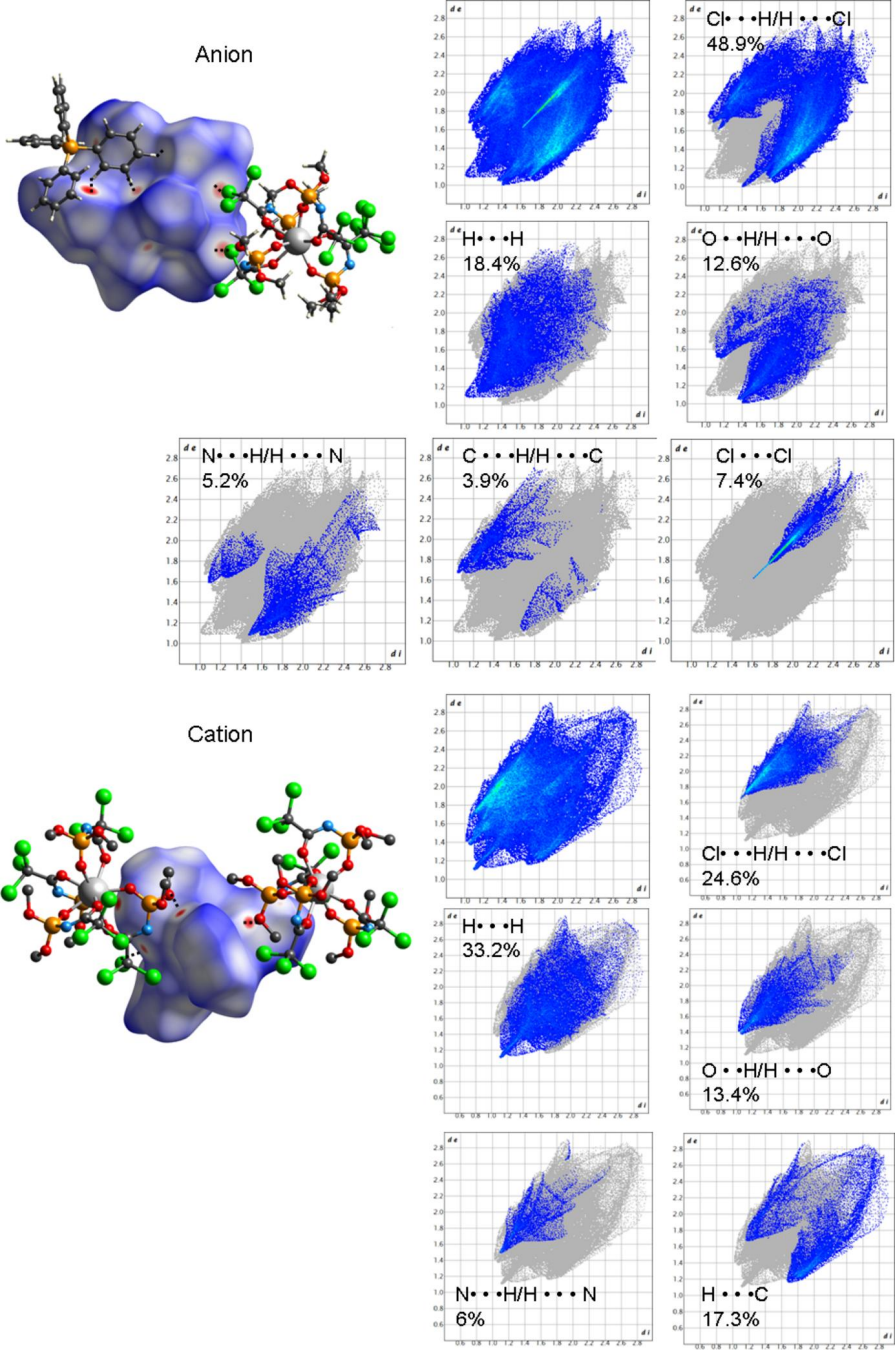
The Hirshfeld surface mapped over *d*_norm_ and two-dimensional fingerprint plots for inter­molecular contacts for the anion and the cation in PPh_4_[Lu*L*_4_].

To visualize the inter­molecular contacts in PPh_4_[Lu*L*_4_], the Hirshfeld surfaces (HS) mapped over *d*_norm_ and the two-dimensional fingerprint plots were generated using *CrystalExplorer 21.5* (Spackman *et al.*, 2021 ▸). Fig. 4 ▸ illustrates the Hirshfeld surfaces for the PPh_4_^+^ cation and the [Lu*L*_4_]^−^ anion. The anion contains oxygen, chlorine, and nitro­gen atoms that act as proton acceptors, forming hydrogen bonds (Table 1 ▸) and making a significant contribution to the inter­molecular inter­actions, in addition to electrostatic attraction between the cations and anions. In contrast, the phenyl groups of the PPh_4_^+^ cation only act as proton donors for hydrogen-bond formation. Weak hydrogen bonds, such as O⋯HC(Ph), Cl⋯HC(Ph) and N⋯HC(Ph), are formed between the cations and anions (Table 1 ▸, Fig. 4 ▸). The regions on the Hirshfeld surface of the cation colored in red correspond to hydrogen bonds of the C—H⋯O, C—H⋯Cl and C—H⋯N (light red) types (Fig. 4 ▸). On the Hirshfeld surface of the complex anion, the red regions represent close contacts between cations and anions, with the most significant inter­actions being inter­molecular hydrogen bonds and C—H⋯O, C—H⋯Cl, C—H⋯N and Cl⋯Cl inter­actions. The figure also shows the atomic contributions (as percentages of the total surface) to the inter­actions between anions and cations. π–π stacking is not observed in the compound. There are six inter­acting anions around the cation and six inter­acting cations around the anion.

## 4. Database survey

Only one structure of a lutetium complex with the carbacyl­amido­phosphate ligand has been reported. It contains the dimethyl (2,2,2-tri­chloro­acet­yl)phospho­ramidate ligand used in the synthesis of PPh_4_[Lu*L*_4_] and has the formula [Lu_2_L_6_·μ-(γ,γ′-dipy)] (refcode QENSIL; Trush *et al.*, 2001 ▸).

A search of the Cambridge Structural Database (CSD, Version 5.44, update of September 2023; Groom *et al.*, 2016 ▸) for compounds containing dimethyl (2,2,2-tri­chloro­acet­yl)phospho­ramidate yielded 21 hits. Dimethyl (2,2,2-tri­chloro­acet­yl)phospho­ramidate forms mono-, bi- and polynuclear coordination compounds with different metals. There are four cases of monodentate coordination of dimethyl (2,2,2-tri­chloro­acet­yl)phospho­ramidate: three *via* oxygen (HATVOO, Trush *et al.*, 2005 ▸; BIGCAV, Trush *et al.*, 1999 ▸; HATWOP, Trush *et al.*, 2005 ▸) and one *via* nitro­gen (VONWUT, Trush *et al.*, 2007 ▸). In the remaining structures, it is coordinated in a bidentate *O*-chelating manner (seven compounds: IHIBUW, Oczko *et al.*, 2003 ▸; QENSIL, Trush *et al.*, 2001 ▸; RUZRIN, Borzechowska *et al.*, 2002 ▸; SAPKIH, Struhatska *et al.*, 2021 ▸; SEMQAF, Yakovlev *et al.*, 2018 ▸; WUKCOV, Znovjyak *et al.*, 2009 ▸; YOFKUA, Puchalska *et al.*, 2008 ▸) or a bridging manner (six compounds: CAPXOG, Bundya *et al.*, 1999 ▸; HATVII, Trush *et al.*, 2005 ▸; HATVUU, Trush *et al.*, 2005 ▸; HATVIJ, Trush *et al.*, 2005 ▸; JAGNUB, Trush *et al.*, 2003 ▸; RUMRIA, Amirkhanov *et al.*, 1996 ▸). Among them, a case of μ-2 coordination *via* oxygen atoms was found (RUMRIA, Amirkhanov *et al.*, 1996 ▸). Another structure contains both a μ-3 bridging ligand connected to the metal *via* oxygen and chlorine and a μ-4 bridging ligand attracting oxygen and nitro­gen atoms for binding to the metal ions (HATVUU, Trush *et al.*, 2005 ▸). The remaining four cases show μ-2 coord­ination involving oxygen atoms of the phosphoryl and carbonyl groups. Among reported H*L*-based compounds there are two complexes of 3d-metals (CAPXOG, Bundya *et al.*, 1999 ▸; JAGNUB, Trush *et al.*, 2003 ▸), four salts of alkaline metals (HATVII, HATVUU and HATWIJ, Trush *et al.*, 2005 ▸; RUMRIA, Amirkhanov *et al.*, 1996 ▸), two of thallium (BIGCAV, Trush *et al.*, 1999 ▸; HATVOO, Trush *et al.*, 2005 ▸), two tetra­phenyl­phospho­nium salts (HATWAB and HATWEF, which also contains a bromide anion and water; Trush *et al.*, 2005 ▸) and one tetra­phenyl­anti­mony(V) salt (HATWOP, Trush *et al.*, 2005 ▸), as well as nine coordination compounds of lanthanides: seven mixed-ligand lanthanide complexes (QENSIL, Trush *et al.*, 2001 ▸; RUZRIN, Borzechowska *et al.*, 2002 ▸; RUZRIN01, Puchalska *et al.*, 2008 ▸; SEMQAF, Yakovlev *et al.*, 2018 ▸; WUKCOV, Znovjyak *et al.*, 2009 ▸; YOFKUA, Puchalska *et al.*, 2008 ▸; IHIBUW, Oczko *et al.*, 2003 ▸) and two *tetra­kis-* CAPh lanthanide complexes Na[Er*L*_4_] and NMe_4_[La*L*_4_] (RUMRIA, Amirkhanov *et al.*, 1996 ▸; SAPKIH, Struhatska *et al.*, 2021 ▸). In the latter two complexes, the ligand is coordinated to the lanthanide ion in a bidentate chelating manner *via* oxygen atoms of the phosphoryl and carbonyl groups. The average *Ln*—O(P) bond lengths are 2.29 and 2.44 Å, respectively, and are shorter than the average Ln—O(C) bond lengths (2.39 and 2.55 Å, respectively). In the structure of the one known lutetium complex {Lu_2_ *L*_6_[μ-(γ, γ′-dipy)]} with H*L* (QENSIL; Trush *et al.*, 2001 ▸), the average Lu—O(P) bond length is 2.22 Å and the average Lu—O(C) bond length is 2.26 Å. The CAPh ligand is coordinated to the lutetium ion in a bidentate chelating manner *via* the PO and CO groups.

## 5. Synthesis and crystallization

**Materials and methods**

Commercially available lutetium nitrate, Lu(NO_3_)_3_·7H_2_O, and tetra­phenyl­phospho­nium bromide, PPh_4_Br, of reagent grade were used in the synthesis. The acetone used was dried and distilled. The ^1^H NMR spectrum of a solution of the title compound in DMSO-*d*_6_ was recorded on a Varian 400 NMR spectrometer at room temperature. The infrared (FT–IR) spectrum was recorded on a Perkin–Elmer BX-II spectrometer using a KBr pellet.

The dimethyl (2,2,2-tri­chloro­acet­yl)phospho­ramidate ligand and its sodium salt were obtained according to a known procedure (Kirsanov *et al.*, 1956 ▸ *)*. The complexes of composition PPh_4_[Ln*L*_4_] with metals La, Nd, Eu, Tb and Y have been synthesized and described previously. The previously used method (Olyshevets *et al.*, 2017 ▸) was adopted for the preparation of the title compound.

**Preparation of PPh_4_[Lu** ***L*** **_4_]**

Lu(NO_3_)_3_·7H_2_O (0.0487 g, 0.1 mmol) in the presence of HC(OC_2_H_5_)_3_ (0.14 ml, 0.7 mmol) as dehydrating agent was dissolved in acetone under heating. In a separate ﬂask, Na*L* (0.1122 g, 0.4 mmol) was dissolved in acetone and PPh_4_Br (0.0419 g, 0.1 mmol) was added under stirring and heating. The two mixtures were combined and boiled for few minutes, then cooled to room temperature. A white precipitate of NaNO_3_ and NaBr was filtered off and the filtrate was left in a ﬂask in a desiccator over CaCl_2_. After two days, colorless crystals suitable for X-ray diffraction studies were obtained. The crystals were ﬁltered off, washed with 2-propanol and dried in air.

IR (KBr pellet, cm^−1^): 2954 [*w*, ν(CH_aliph_)], 1622 [*s*, ν(CO)], 1438 (*w*), 1358 [*s*, ν(CN)], 1164 [*s*, ν(PO)], 1042 [*s*, δ(POC)], 1004 (*m*), 888 (s), 842 (*m*), 820 (*m*), 790 (*w*), 730 (*m*), 674 [*m*, ν(CCl)], 556 [*m*, δ(PNC)], 502 (*m*).

^1^H NMR (400 MHz, DMSO-*d*_6_, 293 K): 3.58, 3.55 (*d*, 24H, CH_3_ [*L*]^−^, *J* = 11.1 Hz), 7.98, 7.97, 7.95 (*t*, 4H, CH [PPh_4_]^+^), 7.82, 7.81, 7.8, 7.79, 7.76, 7.75, 7.73, 7.71 (*m*, 16H, CH [PPh_4_]^+^).

A comparison of the IR spectra of the obtained compound with the spectra of the ligand and of its sodium salt was carried out. In the IR spectrum of PPh_4_[Lu*L*_4_], characteristic absorption bands of the carbonyl and phosphoryl groups are observed at 1622 and 1164 cm^−1^, respectively. There is a noticeable shift of the absorption bands of the carbonyl and phosphoryl groups in the spectrum of the complex towards lower wavenumbers compared to the spectra of the free ligand (110 and 104 cm^−1^, respectively) and the sodium salt (2 and 36 cm^−1^, respectively). This is consistent with the observed lengthening of the P=O and C=O bond lengths in the structure when compared to the ligand and sodium salt structures. The absorption band ν(N—H), which is observed in the IR spectrum of H*L* at 3080 cm^−1^, is absent in the IR spectrum of the PPh_4_[Lu*L*_4_] complex, indicating ligand coordination in the deprotonated form. The presence of the tetra­phenyl­phospho­nium cation in the complex can be confirmed by the IR spectrum, showing bands at 1439, 1108, and 528 cm^−1^, which are absent in the IR spectrum of Na*L*.

## 6. Refinement

Crystal data, data collection and structure refinement details are summarized in Table 2 ▸. The hydrogen-atom positions were positioned geometrically (C—H = 0.95–0.98 Å) refined using a riding model, with fixed *U*_iso_ values of 1.2*U*_iso_ of the attached C atom for aromatic H atoms and 1.5 for CH_3_ groups. The methyl group was refined as a rotating group. One of the phosphoryl ligands is disordered. The chlorine atoms of the CCl_3_ group and the CH_3_ group of the meth­oxy substituents refined to occupancy ratios of 0.868 (3):0.132 (3) and 0.62 (5):0.38 (5). The major component of the disordered CCl_3_ group was refined in an anisotropic approximation, while the minor component was refined isotropically. Additionally, some C—Cl distances were restrained to 1.750 Å with a sigma value of 0.001.

## Supplementary Material

Crystal structure: contains datablock(s) I. DOI: 10.1107/S205698902400210X/dj2075sup1.cif

Structure factors: contains datablock(s) I. DOI: 10.1107/S205698902400210X/dj2075Isup3.hkl

CCDC reference: 2337153

Additional supporting information:  crystallographic information; 3D view; checkCIF report

## Figures and Tables

**Table 1:** Hydrogen-bond geometry (Å, °)

*D*—H⋯*A*	*D*—H	H⋯*A*	*D*⋯*A*	*D*—H⋯*A*
C26—H26*A*⋯O44	0.95	2.56	3.402 (6)	148
C14—H14*B*⋯O22	0.98	2.61	3.561 (5)	164
C43*B*—H43*E*⋯Cl12	0.98	2.93	3.73 (6)	140
C20—H20*A*⋯Cl1*A*	0.95	2.83	3.694 (5)	152
C34—H34*B*⋯O31	0.98	2.36	2.887 (6)	113
C19—H19*A*⋯O21	0.95	2.56	3.479 (6)	162
C43*B*—H43*D*⋯Cl32^i^	0.98	2.86	3.37 (2)	113
C40—H40*A*⋯Cl11^ii^	0.95	2.94	3.676 (4)	135
C23—H23*A*⋯Cl3*A* ^iii^	0.98	2.99	3.768 (5)	137
C33—H33*A*⋯Cl33^iv^	0.98	2.89	3.597 (5)	130
C50—H50*A*⋯O23^v^	0.95	2.56	3.355 (5)	142
C49—H49*A*⋯N2^v^	0.95	2.71	3.540 (6)	146
C49—H49*A*⋯Cl21^v^	0.95	2.99	3.795 (5)	143
C17—H17*A*⋯O33^vi^	0.95	2.88	3.383 (6)	114
C17—H17*A*⋯N3^vi^	0.95	2.68	3.424 (6)	136

Symmetry codes: (i) 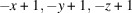 ; (ii) 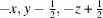 ; (iii) 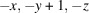 ; (iv) 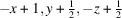 ; (v) 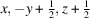 ; (vi) 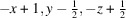 .

**Table 2:** Experimental details

Crystal data	
Chemical formula	(C_24_H_20_)[Lu(C_4_H_6_Cl_3_NO_4_P)_4_]
*M*_r_	1592.01
Crystal system, space group	Monoclinic, *P*2_1_/*c*
Temperature (K)	100
*a*, *b*, *c* (Å)	19.6882 (3), 18.9452 (10), 17.2139 (3)
β (°)	110.8107 (15)
*V* (Å^3^)	6001.8 (3)
*Z*	4
Radiation type	Cu *K*α
μ (mm^−1^)	9.89
Crystal size (mm)	0.3 × 0.3 × 0.3

Data collection	
Diffractometer	Rigaku XtaLAB Synergy R with HyPix-Arc 150
Absorption correction	Multi-scan (*CrysAlis PRO*; Rigaku OD, 2022 ▸)
*T*_min_, *T*_max_	0.513, 1.000
No. of measured, independent and observed [*I* > 2σ(*I*)] reflections	68030, 11789, 10535
*R*_int_	0.059
(sin θ/λ)_max_ (Å^−1^)	0.623

Refinement	
*R*[*F* ^2^ > 2σ(*F* ^2^)], *wR*(*F* ^2^), *S*	0.040, 0.108, 1.08
No. of reflections	11789
No. of parameters	735
No. of restraints	7
H-atom treatment	H-atom parameters constrained
Δρ_max_, Δρ_min_ (e Å^−3^)	1.32, −1.07

Computer programs: *CrysAlis PRO* (Rigaku OD, 2022 ▸), *SHELXS* (Sheldrick, 2008 ▸), *SHELXL2019/2* (Sheldrick, 2015 ▸) and *ORTEPIII* for Windows (Farrugia, 2012 ▸).

## supplementary crystallographic information

### Crystal data

**Table:**

(C_24_H_20_P)[Lu(C_4_H_6_Cl_3_NO_4_P)_4_]	*F*(000) = 3160
*M**_r_* = 1592.01	*D*_x_ = 1.762 Mg m^−^^3^
Monoclinic, *P*2_1_/*c*	Cu *K*α radiation, λ = 1.54184 Å
*a* = 19.6882 (3) Å	Cell parameters from 36128 reflections
*b* = 18.9452 (10) Å	θ = 2.4–73.8°
*c* = 17.2139 (3) Å	µ = 9.89 mm^−^^1^
β = 110.8107 (15)°	*T* = 100 K
*V* = 6001.8 (3) Å^3^	Block, colorless
*Z* = 4	0.3 × 0.3 × 0.3 mm

### Data collection

**Table:**

Rigaku XtaLAB Synergy R with HyPix-Arc 150 diffractometer	11789 independent reflections
Radiation source: Rotating-anode X-ray tube, Rigaku (Cu) X-ray Source	10535 reflections with *I* > 2σ(*I*)
Detector resolution: 10.0000 pixels mm^-1^	*R*_int_ = 0.059
ω scans	θ_max_ = 73.9°, θ_min_ = 2.4°
Absorption correction: multi-scan (CrysAlisPro; Rigaku OD, 2022)	*h* = −24→24
*T*_min_ = 0.513, *T*_max_ = 1.000	*k* = −23→23
68030 measured reflections	*l* = −17→21

### Refinement

**Table:**

Refinement on *F*^2^	7 restraints
Least-squares matrix: full	Hydrogen site location: inferred from neighbouring sites
*R*[*F*^2^ > 2σ(*F*^2^)] = 0.040	H-atom parameters constrained
*wR*(*F*^2^) = 0.108	*w* = 1/[σ^2^(*F*_o_^2^) + (0.0532*P*)^2^ + 11.4066*P*] where *P* = (*F*_o_^2^ + 2*F*_c_^2^)/3
*S* = 1.08	(Δ/σ)_max_ = 0.002
11789 reflections	Δρ_max_ = 1.32 e Å^−^^3^
735 parameters	Δρ_min_ = −1.07 e Å^−^^3^

### Special details

**Table:**

Experimental. X-ray analyses of PPh_4_[Lu*L*_4_] were performed on an XtaLAB Synergy R, Dual Wavelength system, using Cu *K*α radiation (λ = 1.5418 Å) and a Hybrid Pixel Array HyPix-Arc 150 detector at 100 K.
Geometry. All esds (except the esd in the dihedral angle between two l.s. planes) are estimated using the full covariance matrix. The cell esds are taken into account individually in the estimation of esds in distances, angles and torsion angles; correlations between esds in cell parameters are only used when they are defined by crystal symmetry. An approximate (isotropic) treatment of cell esds is used for estimating esds involving l.s. planes.

### Fractional atomic coordinates and isotropic or equivalent isotropic displacement parameters (Å^2^)

**Table:**

	*x*	*y*	*z*	*U*_iso_*/*U*_eq_	Occ. (<1)
Lu1	0.30812 (2)	0.52340 (2)	0.24117 (2)	0.02160 (7)	
Cl1A	0.09212 (6)	0.36510 (8)	0.07379 (7)	0.0405 (4)	0.868 (3)
Cl2A	0.03572 (7)	0.34419 (9)	0.20331 (7)	0.0513 (5)	0.868 (3)
Cl3A	0.03339 (8)	0.48308 (8)	0.13322 (16)	0.0625 (6)	0.868 (3)
Cl1B	0.0709 (13)	0.3239 (6)	0.1084 (12)	0.133 (8)*	0.132 (3)
Cl2B	0.0208 (5)	0.4021 (7)	0.2140 (7)	0.071 (4)*	0.132 (3)
Cl3B	0.0433 (8)	0.4763 (6)	0.0972 (8)	0.075 (5)*	0.132 (3)
C43A	0.3208 (6)	0.4020 (17)	0.5146 (8)	0.052 (4)	0.62 (5)
H43A	0.306414	0.417250	0.560985	0.078*	0.62 (5)
H43B	0.356357	0.435244	0.507796	0.078*	0.62 (5)
H43C	0.342436	0.354796	0.526273	0.078*	0.62 (5)
C43B	0.3050 (19)	0.440 (3)	0.4999 (15)	0.057 (9)	0.38 (5)
H43D	0.288628	0.443234	0.547341	0.085*	0.38 (5)
H43E	0.307662	0.487720	0.478709	0.085*	0.38 (5)
H43F	0.353186	0.418203	0.517715	0.085*	0.38 (5)
P3	0.46486 (5)	0.60714 (5)	0.36566 (6)	0.0262 (2)	
O32	0.42365 (14)	0.47177 (14)	0.27693 (17)	0.0262 (6)	
O42	0.18959 (15)	0.47307 (15)	0.20058 (17)	0.0311 (6)	
N2	0.3317 (2)	0.52389 (18)	0.0298 (2)	0.0322 (8)	
O12	0.24928 (15)	0.57523 (15)	0.32441 (16)	0.0311 (6)	
O11	0.23433 (14)	0.60386 (14)	0.15668 (16)	0.0274 (6)	
O41	0.31681 (14)	0.44195 (14)	0.33823 (16)	0.0259 (5)	
O24	0.34595 (17)	0.38773 (16)	0.04136 (18)	0.0370 (7)	
C12	0.1787 (2)	0.6328 (2)	0.3905 (3)	0.0370 (10)	
O33	0.49700 (17)	0.68190 (16)	0.35963 (19)	0.0376 (7)	
O23	0.22456 (16)	0.43826 (16)	−0.01704 (17)	0.0348 (6)	
O34	0.48780 (16)	0.59568 (18)	0.46207 (17)	0.0398 (7)	
O13	0.11413 (17)	0.67104 (18)	0.10392 (19)	0.0419 (7)	
C19	0.2909 (3)	0.2636 (3)	0.1581 (4)	0.0632 (16)	
H19A	0.286095	0.311037	0.139117	0.076*	
O22	0.36999 (14)	0.57898 (14)	0.15909 (15)	0.0250 (5)	
C31	0.4859 (2)	0.4967 (2)	0.2957 (2)	0.0263 (8)	
C40	0.0260 (2)	0.1450 (2)	0.0609 (3)	0.0361 (9)	
H40A	0.009692	0.157753	0.104700	0.043*	
O31	0.38470 (14)	0.60400 (14)	0.32491 (16)	0.0272 (6)	
O14	0.22822 (17)	0.73667 (16)	0.15450 (19)	0.0386 (7)	
C15	0.2386 (2)	0.1577 (2)	0.1887 (3)	0.0328 (9)	
O21	0.29546 (15)	0.44604 (14)	0.13784 (16)	0.0283 (6)	
C21	0.3629 (2)	0.5727 (2)	0.0853 (2)	0.0261 (8)	
C13	0.1034 (3)	0.6581 (3)	0.0180 (3)	0.0495 (12)	
H13A	0.064326	0.688502	−0.017253	0.074*	
H13B	0.090173	0.608526	0.004732	0.074*	
H13C	0.148406	0.668492	0.008115	0.074*	
O44	0.30205 (17)	0.31240 (16)	0.3592 (2)	0.0410 (7)	
C16	0.3064 (2)	0.1258 (3)	0.2175 (3)	0.0417 (10)	
H16A	0.311920	0.079182	0.239017	0.050*	
C20	0.2308 (3)	0.2267 (3)	0.1595 (4)	0.0506 (13)	
H20A	0.184328	0.248461	0.140523	0.061*	
C38	0.0040 (3)	0.1343 (3)	−0.0851 (3)	0.0423 (10)	
H38A	−0.027983	0.138171	−0.141144	0.051*	
C47	0.1889 (2)	−0.1020 (2)	0.1770 (3)	0.0359 (9)	
H47A	0.182281	−0.138391	0.137095	0.043*	
Cl31	0.52815 (8)	0.48361 (9)	0.16518 (7)	0.0614 (4)	
C39	−0.0198 (3)	0.1517 (3)	−0.0213 (3)	0.0442 (11)	
H39A	−0.068044	0.168384	−0.033791	0.053*	
C32	0.5380 (2)	0.4514 (2)	0.2654 (3)	0.0309 (8)	
N3	0.51473 (17)	0.55532 (18)	0.3336 (2)	0.0289 (7)	
C41	0.1615 (2)	0.4226 (2)	0.2240 (2)	0.0280 (8)	
C34	0.4372 (3)	0.5806 (3)	0.5022 (3)	0.0547 (14)	
H34A	0.447691	0.533922	0.528283	0.082*	
H34B	0.387764	0.581141	0.461067	0.082*	
H34C	0.441577	0.616397	0.544800	0.082*	
Cl32	0.62997 (5)	0.45770 (6)	0.32937 (7)	0.0381 (2)	
Cl23	0.45983 (6)	0.68373 (7)	0.12537 (7)	0.0464 (3)	
P4	0.26757 (5)	0.38843 (5)	0.35292 (6)	0.0267 (2)	
Cl21	0.31894 (7)	0.69263 (6)	−0.00345 (7)	0.0450 (3)	
P2	0.30013 (5)	0.45186 (5)	0.05381 (6)	0.0265 (2)	
Cl22	0.43061 (8)	0.60520 (6)	−0.02568 (8)	0.0496 (3)	
Cl11	0.12830 (8)	0.70995 (6)	0.38663 (9)	0.0555 (3)	
P1	0.19335 (6)	0.66579 (6)	0.17026 (7)	0.0312 (2)	
Cl12	0.25254 (7)	0.63051 (9)	0.48617 (7)	0.0588 (4)	
P5	0.15805 (5)	0.11087 (5)	0.18373 (6)	0.0269 (2)	
Cl33	0.51374 (7)	0.36110 (6)	0.25932 (10)	0.0578 (4)	
C17	0.3661 (3)	0.1630 (3)	0.2143 (4)	0.0593 (15)	
H17A	0.412707	0.141448	0.232801	0.071*	
Cl13	0.12303 (6)	0.55772 (6)	0.38694 (7)	0.0401 (2)	
O43	0.25654 (16)	0.39996 (18)	0.43804 (17)	0.0384 (7)	
C27	0.1282 (3)	0.2266 (3)	0.3676 (3)	0.0498 (12)	
H27A	0.152949	0.262532	0.405660	0.060*	
C24	0.3597 (3)	0.3797 (3)	−0.0347 (3)	0.0517 (13)	
H24A	0.400407	0.347059	−0.025809	0.078*	
H24B	0.371812	0.425675	−0.052334	0.078*	
H24C	0.316239	0.360759	−0.077846	0.078*	
C18	0.3581 (3)	0.2309 (3)	0.1845 (4)	0.0697 (19)	
H18A	0.399159	0.255730	0.181982	0.084*	
C22	0.3937 (2)	0.6348 (2)	0.0479 (3)	0.0349 (9)	
C37	0.0749 (3)	0.1112 (2)	−0.0673 (3)	0.0399 (10)	
H37A	0.091711	0.100427	−0.111267	0.048*	
C42	0.0823 (2)	0.40435 (13)	0.16142 (14)	0.0338 (9)	
C35	0.0956 (2)	0.1197 (2)	0.0784 (2)	0.0295 (8)	
C11	0.2067 (2)	0.6251 (2)	0.3161 (3)	0.0304 (8)	
C33	0.4738 (3)	0.7416 (2)	0.3975 (3)	0.0421 (11)	
H33A	0.498876	0.784439	0.390338	0.063*	
H33B	0.485591	0.732526	0.456890	0.063*	
H33C	0.421165	0.748114	0.370676	0.063*	
N1	0.1799 (2)	0.6714 (2)	0.2578 (2)	0.0378 (8)	
N4	0.18567 (18)	0.38005 (19)	0.2877 (2)	0.0323 (7)	
C26	0.1568 (3)	0.2016 (2)	0.3094 (3)	0.0401 (10)	
H26A	0.199860	0.221571	0.305818	0.048*	
C36	0.1206 (2)	0.1040 (2)	0.0137 (3)	0.0356 (9)	
H36A	0.169085	0.088357	0.025862	0.043*	
C45	0.1794 (2)	0.0201 (2)	0.2100 (3)	0.0293 (8)	
C25	0.1212 (2)	0.1468 (2)	0.2567 (3)	0.0291 (8)	
C46	0.1695 (2)	−0.0331 (2)	0.1508 (3)	0.0326 (9)	
H46A	0.149630	−0.022265	0.093221	0.039*	
C30	0.0579 (2)	0.1178 (2)	0.2615 (3)	0.0370 (10)	
H30A	0.034868	0.079325	0.226658	0.044*	
C28	0.0641 (3)	0.1992 (3)	0.3701 (3)	0.0475 (12)	
H28A	0.044067	0.217793	0.408527	0.057*	
C48	0.2175 (3)	−0.1178 (3)	0.2604 (3)	0.0437 (11)	
H48A	0.228090	−0.165482	0.277662	0.052*	
C29	0.0285 (3)	0.1450 (3)	0.3172 (3)	0.0441 (11)	
H29A	−0.015810	0.126713	0.319146	0.053*	
C14	0.3054 (3)	0.7467 (3)	0.1957 (3)	0.0460 (11)	
H14A	0.320509	0.789805	0.174748	0.069*	
H14B	0.331454	0.706144	0.184517	0.069*	
H14C	0.316698	0.751020	0.255688	0.069*	
C50	0.2109 (3)	0.0040 (3)	0.2937 (3)	0.0441 (11)	
H50A	0.218762	0.040381	0.333944	0.053*	
C49	0.2311 (3)	−0.0648 (3)	0.3195 (3)	0.0508 (13)	
H49A	0.254012	−0.075361	0.376863	0.061*	
C23	0.1674 (3)	0.4876 (3)	−0.0238 (3)	0.0471 (12)	
H23A	0.121274	0.469135	−0.062383	0.071*	
H23B	0.178200	0.532806	−0.044585	0.071*	
H23C	0.163766	0.494584	0.031007	0.071*	
C44	0.3783 (3)	0.3044 (3)	0.3774 (5)	0.0712 (19)	
H44A	0.389015	0.255147	0.368709	0.107*	
H44B	0.404772	0.317577	0.435429	0.107*	
H44C	0.393517	0.335028	0.340694	0.107*	

### Atomic displacement parameters (Å^2^)

**Table:**

	*U*^11^	*U*^22^	*U*^33^	*U*^12^	*U*^13^	*U*^23^
Lu1	0.02115 (10)	0.02505 (11)	0.02073 (10)	−0.00046 (7)	0.01006 (7)	−0.00134 (7)
Cl1A	0.0320 (6)	0.0588 (8)	0.0284 (6)	−0.0052 (5)	0.0078 (4)	−0.0129 (5)
Cl2A	0.0338 (7)	0.0854 (12)	0.0309 (6)	−0.0266 (7)	0.0068 (5)	0.0069 (6)
Cl3A	0.0265 (7)	0.0439 (8)	0.0990 (15)	0.0123 (5)	0.0001 (8)	−0.0143 (8)
C43A	0.035 (5)	0.098 (13)	0.022 (5)	−0.004 (6)	0.010 (4)	−0.007 (6)
C43B	0.050 (13)	0.10 (2)	0.028 (8)	−0.027 (14)	0.018 (8)	−0.009 (11)
P3	0.0241 (5)	0.0322 (5)	0.0251 (5)	−0.0023 (4)	0.0122 (4)	−0.0077 (4)
O32	0.0239 (13)	0.0268 (14)	0.0304 (14)	0.0010 (10)	0.0127 (11)	−0.0002 (11)
O42	0.0266 (14)	0.0389 (16)	0.0280 (14)	−0.0066 (12)	0.0098 (11)	0.0010 (12)
N2	0.044 (2)	0.0349 (19)	0.0246 (16)	−0.0058 (15)	0.0205 (15)	−0.0026 (14)
O12	0.0304 (14)	0.0394 (16)	0.0289 (14)	0.0037 (12)	0.0173 (11)	−0.0036 (12)
O11	0.0269 (13)	0.0276 (14)	0.0282 (13)	0.0031 (11)	0.0106 (11)	0.0023 (11)
O41	0.0250 (13)	0.0280 (14)	0.0268 (13)	−0.0045 (11)	0.0115 (10)	0.0027 (11)
O24	0.0471 (18)	0.0345 (16)	0.0330 (15)	0.0067 (13)	0.0185 (13)	−0.0024 (12)
C12	0.036 (2)	0.037 (2)	0.047 (2)	−0.0037 (18)	0.025 (2)	−0.0111 (19)
O33	0.0411 (17)	0.0325 (15)	0.0485 (17)	−0.0087 (13)	0.0274 (14)	−0.0149 (13)
O23	0.0378 (16)	0.0391 (16)	0.0259 (13)	−0.0037 (13)	0.0095 (12)	−0.0040 (12)
O34	0.0335 (15)	0.062 (2)	0.0239 (14)	0.0013 (15)	0.0105 (12)	−0.0060 (13)
O13	0.0336 (16)	0.0481 (19)	0.0436 (17)	0.0081 (14)	0.0133 (13)	0.0067 (15)
C19	0.071 (4)	0.047 (3)	0.088 (4)	−0.018 (3)	0.048 (3)	0.001 (3)
O22	0.0261 (13)	0.0276 (13)	0.0236 (12)	−0.0026 (11)	0.0117 (10)	−0.0009 (10)
C31	0.0262 (19)	0.033 (2)	0.0235 (18)	0.0015 (16)	0.0137 (15)	−0.0003 (16)
C40	0.030 (2)	0.046 (3)	0.037 (2)	−0.0005 (19)	0.0184 (18)	−0.0008 (19)
O31	0.0257 (13)	0.0301 (14)	0.0265 (13)	−0.0015 (11)	0.0101 (11)	−0.0107 (11)
O14	0.0423 (17)	0.0304 (15)	0.0460 (17)	0.0007 (13)	0.0191 (14)	0.0014 (13)
C15	0.0262 (19)	0.033 (2)	0.044 (2)	−0.0043 (17)	0.0188 (18)	−0.0054 (18)
O21	0.0372 (15)	0.0238 (13)	0.0271 (13)	−0.0045 (11)	0.0152 (11)	−0.0030 (11)
C21	0.0284 (19)	0.0260 (19)	0.0283 (19)	0.0010 (15)	0.0156 (15)	0.0024 (15)
C13	0.038 (3)	0.062 (3)	0.041 (3)	−0.001 (2)	0.004 (2)	0.012 (2)
O44	0.0386 (17)	0.0305 (16)	0.0494 (18)	−0.0038 (13)	0.0103 (14)	0.0004 (14)
C16	0.035 (2)	0.041 (2)	0.054 (3)	0.0012 (19)	0.022 (2)	−0.011 (2)
C20	0.042 (3)	0.038 (3)	0.080 (4)	−0.002 (2)	0.031 (3)	0.006 (2)
C38	0.042 (3)	0.048 (3)	0.036 (2)	0.001 (2)	0.014 (2)	0.001 (2)
C47	0.043 (2)	0.028 (2)	0.035 (2)	−0.0015 (18)	0.0112 (18)	−0.0069 (17)
Cl31	0.0661 (8)	0.0943 (11)	0.0340 (6)	0.0326 (8)	0.0301 (6)	0.0042 (6)
C39	0.033 (2)	0.059 (3)	0.044 (3)	0.004 (2)	0.017 (2)	0.001 (2)
C32	0.0235 (19)	0.035 (2)	0.036 (2)	0.0007 (16)	0.0128 (16)	−0.0083 (18)
N3	0.0226 (15)	0.0347 (18)	0.0311 (17)	−0.0026 (14)	0.0116 (13)	−0.0096 (14)
C41	0.0231 (18)	0.038 (2)	0.0265 (18)	−0.0052 (16)	0.0130 (15)	−0.0035 (16)
C34	0.057 (3)	0.079 (4)	0.036 (2)	0.004 (3)	0.026 (2)	0.005 (3)
Cl32	0.0244 (5)	0.0423 (6)	0.0489 (6)	0.0026 (4)	0.0147 (4)	−0.0055 (5)
Cl23	0.0473 (6)	0.0497 (6)	0.0477 (6)	−0.0208 (5)	0.0236 (5)	−0.0074 (5)
P4	0.0257 (5)	0.0309 (5)	0.0236 (4)	−0.0058 (4)	0.0090 (4)	−0.0002 (4)
Cl21	0.0603 (7)	0.0313 (5)	0.0444 (6)	0.0030 (5)	0.0197 (5)	0.0061 (4)
P2	0.0324 (5)	0.0267 (5)	0.0221 (4)	−0.0016 (4)	0.0117 (4)	−0.0025 (4)
Cl22	0.0750 (8)	0.0408 (6)	0.0563 (7)	−0.0076 (6)	0.0518 (7)	−0.0036 (5)
Cl11	0.0743 (9)	0.0363 (6)	0.0838 (9)	0.0015 (6)	0.0624 (8)	−0.0098 (6)
P1	0.0307 (5)	0.0289 (5)	0.0377 (5)	0.0043 (4)	0.0168 (4)	0.0022 (4)
Cl12	0.0466 (7)	0.0989 (11)	0.0363 (6)	−0.0137 (7)	0.0214 (5)	−0.0248 (6)
P5	0.0251 (5)	0.0239 (5)	0.0350 (5)	−0.0003 (4)	0.0148 (4)	−0.0009 (4)
Cl33	0.0422 (6)	0.0358 (6)	0.1030 (11)	−0.0048 (5)	0.0352 (7)	−0.0249 (6)
C17	0.034 (3)	0.064 (4)	0.089 (4)	−0.010 (2)	0.033 (3)	−0.024 (3)
Cl13	0.0374 (5)	0.0375 (5)	0.0553 (6)	−0.0033 (4)	0.0288 (5)	−0.0018 (5)
O43	0.0329 (15)	0.056 (2)	0.0278 (14)	−0.0135 (14)	0.0127 (12)	−0.0029 (13)
C27	0.069 (3)	0.036 (3)	0.054 (3)	−0.005 (2)	0.034 (3)	−0.014 (2)
C24	0.062 (3)	0.059 (3)	0.042 (3)	0.019 (3)	0.029 (2)	−0.005 (2)
C18	0.058 (4)	0.065 (4)	0.110 (5)	−0.028 (3)	0.058 (4)	−0.025 (4)
C22	0.047 (2)	0.030 (2)	0.036 (2)	−0.0040 (18)	0.025 (2)	−0.0032 (17)
C37	0.051 (3)	0.040 (2)	0.038 (2)	0.003 (2)	0.027 (2)	0.0028 (19)
C42	0.026 (2)	0.045 (2)	0.030 (2)	−0.0038 (18)	0.0100 (16)	0.0006 (18)
C35	0.028 (2)	0.029 (2)	0.033 (2)	0.0018 (16)	0.0138 (16)	0.0035 (16)
C11	0.0274 (19)	0.035 (2)	0.034 (2)	−0.0052 (17)	0.0179 (17)	−0.0082 (18)
C33	0.047 (3)	0.035 (2)	0.046 (3)	−0.001 (2)	0.018 (2)	−0.015 (2)
N1	0.040 (2)	0.036 (2)	0.046 (2)	0.0044 (16)	0.0254 (17)	−0.0050 (16)
N4	0.0270 (17)	0.0395 (19)	0.0287 (17)	−0.0085 (15)	0.0076 (14)	0.0042 (15)
C26	0.044 (3)	0.035 (2)	0.049 (3)	−0.0058 (19)	0.026 (2)	−0.010 (2)
C36	0.035 (2)	0.038 (2)	0.040 (2)	0.0021 (18)	0.0205 (19)	0.0043 (19)
C45	0.032 (2)	0.0245 (19)	0.033 (2)	0.0013 (16)	0.0127 (17)	−0.0016 (16)
C25	0.030 (2)	0.0250 (19)	0.036 (2)	0.0004 (16)	0.0161 (17)	−0.0017 (16)
C46	0.038 (2)	0.029 (2)	0.030 (2)	0.0029 (17)	0.0116 (17)	−0.0005 (16)
C30	0.040 (2)	0.039 (2)	0.038 (2)	−0.0034 (19)	0.0219 (19)	−0.0003 (19)
C28	0.071 (3)	0.038 (3)	0.052 (3)	0.009 (2)	0.044 (3)	0.002 (2)
C48	0.063 (3)	0.034 (2)	0.039 (2)	0.013 (2)	0.024 (2)	0.0066 (19)
C29	0.046 (3)	0.048 (3)	0.050 (3)	−0.002 (2)	0.030 (2)	0.003 (2)
C14	0.042 (3)	0.035 (2)	0.065 (3)	−0.003 (2)	0.024 (2)	−0.009 (2)
C50	0.065 (3)	0.038 (2)	0.030 (2)	0.006 (2)	0.017 (2)	−0.0065 (19)
C49	0.082 (4)	0.040 (3)	0.031 (2)	0.021 (3)	0.021 (2)	0.006 (2)
C23	0.040 (3)	0.063 (3)	0.036 (2)	0.008 (2)	0.010 (2)	0.000 (2)
C44	0.044 (3)	0.041 (3)	0.114 (5)	0.006 (2)	0.010 (3)	−0.016 (3)

### Geometric parameters (Å, º)

**Table:**

Lu1—O41	2.236 (3)	C20—H20A	0.9500
Lu1—O11	2.247 (3)	C38—C39	1.378 (6)
Lu1—O21	2.249 (3)	C38—C37	1.388 (7)
Lu1—O31	2.267 (3)	C38—H38A	0.9500
Lu1—O32	2.348 (3)	C47—C48	1.376 (6)
Lu1—O12	2.353 (3)	C47—C46	1.389 (6)
Lu1—O42	2.384 (3)	C47—H47A	0.9500
Lu1—O22	2.411 (2)	Cl31—C32	1.775 (4)
Cl1A—C42	1.7529 (10)	C39—H39A	0.9500
Cl2A—C42	1.769 (3)	C32—Cl32	1.758 (4)
Cl3A—C42	1.748 (3)	C32—Cl33	1.768 (4)
Cl1B—C42	1.7495 (10)	C41—N4	1.306 (5)
Cl2B—C42	1.7508 (10)	C41—C42	1.586 (5)
Cl3B—C42	1.7508 (10)	C34—H34A	0.9800
C43A—O43	1.468 (13)	C34—H34B	0.9800
C43A—H43A	0.9800	C34—H34C	0.9800
C43A—H43B	0.9800	Cl23—C22	1.761 (5)
C43A—H43C	0.9800	P4—O43	1.571 (3)
C43B—O43	1.38 (2)	P4—N4	1.613 (3)
C43B—H43D	0.9800	Cl21—C22	1.795 (5)
C43B—H43E	0.9800	Cl22—C22	1.761 (4)
C43B—H43F	0.9800	P1—N1	1.624 (4)
P3—O31	1.483 (3)	P5—C45	1.789 (4)
P3—O33	1.570 (3)	P5—C25	1.793 (4)
P3—O34	1.573 (3)	P5—C35	1.801 (4)
P3—N3	1.618 (3)	C17—C18	1.372 (9)
O32—C31	1.245 (5)	C17—H17A	0.9500
O42—C41	1.242 (5)	C27—C28	1.379 (7)
N2—C21	1.315 (5)	C27—C26	1.395 (6)
N2—P2	1.613 (3)	C27—H27A	0.9500
O12—C11	1.238 (5)	C24—H24A	0.9800
O11—P1	1.489 (3)	C24—H24B	0.9800
O41—P4	1.485 (3)	C24—H24C	0.9800
O24—C24	1.436 (5)	C18—H18A	0.9500
O24—P2	1.573 (3)	C37—C36	1.371 (6)
C12—C11	1.569 (5)	C37—H37A	0.9500
C12—Cl11	1.755 (5)	C35—C36	1.400 (6)
C12—Cl12	1.768 (5)	C11—N1	1.295 (6)
C12—Cl13	1.784 (5)	C33—H33A	0.9800
O33—C33	1.459 (5)	C33—H33B	0.9800
O23—C23	1.435 (6)	C33—H33C	0.9800
O23—P2	1.574 (3)	C26—C25	1.394 (6)
O34—C34	1.428 (6)	C26—H26A	0.9500
O13—C13	1.438 (6)	C36—H36A	0.9500
O13—P1	1.575 (3)	C45—C50	1.386 (6)
C19—C20	1.381 (7)	C45—C46	1.396 (6)
C19—C18	1.383 (9)	C25—C30	1.392 (6)
C19—H19A	0.9500	C46—H46A	0.9500
O22—C21	1.233 (4)	C30—C29	1.383 (6)
C31—N3	1.310 (5)	C30—H30A	0.9500
C31—C32	1.562 (5)	C28—C29	1.387 (7)
C40—C35	1.380 (6)	C28—H28A	0.9500
C40—C39	1.389 (6)	C48—C49	1.386 (7)
C40—H40A	0.9500	C48—H48A	0.9500
O14—C14	1.443 (6)	C29—H29A	0.9500
O14—P1	1.575 (3)	C14—H14A	0.9800
C15—C16	1.386 (6)	C14—H14B	0.9800
C15—C20	1.389 (7)	C14—H14C	0.9800
C15—P5	1.793 (4)	C50—C49	1.388 (7)
O21—P2	1.486 (3)	C50—H50A	0.9500
C21—C22	1.564 (5)	C49—H49A	0.9500
C13—H13A	0.9800	C23—H23A	0.9800
C13—H13B	0.9800	C23—H23B	0.9800
C13—H13C	0.9800	C23—H23C	0.9800
O44—C44	1.429 (6)	C44—H44A	0.9800
O44—P4	1.580 (3)	C44—H44B	0.9800
C16—C17	1.388 (7)	C44—H44C	0.9800
C16—H16A	0.9500		
			
O41—Lu1—O11	144.31 (9)	O43—P4—N4	103.19 (17)
O41—Lu1—O21	95.67 (10)	O44—P4—N4	104.89 (18)
O11—Lu1—O21	93.77 (10)	O21—P2—O24	108.19 (16)
O41—Lu1—O31	97.62 (10)	O21—P2—O23	111.94 (16)
O11—Lu1—O31	94.91 (10)	O24—P2—O23	101.42 (17)
O21—Lu1—O31	143.48 (9)	O21—P2—N2	118.37 (16)
O41—Lu1—O32	72.95 (9)	O24—P2—N2	109.09 (18)
O11—Lu1—O32	142.71 (9)	O23—P2—N2	106.48 (18)
O21—Lu1—O32	76.16 (10)	O11—P1—O14	110.56 (16)
O31—Lu1—O32	75.55 (9)	O11—P1—O13	112.85 (17)
O41—Lu1—O12	75.91 (10)	O14—P1—O13	101.61 (18)
O11—Lu1—O12	76.47 (10)	O11—P1—N1	118.28 (18)
O21—Lu1—O12	144.40 (10)	O14—P1—N1	109.21 (19)
O31—Lu1—O12	72.05 (9)	O13—P1—N1	102.84 (19)
O32—Lu1—O12	130.89 (9)	C45—P5—C25	107.76 (19)
O41—Lu1—O42	75.53 (9)	C45—P5—C15	109.8 (2)
O11—Lu1—O42	75.00 (10)	C25—P5—C15	110.48 (19)
O21—Lu1—O42	71.68 (10)	C45—P5—C35	111.05 (19)
O31—Lu1—O42	144.72 (9)	C25—P5—C35	111.61 (19)
O32—Lu1—O42	131.78 (10)	C15—P5—C35	106.1 (2)
O12—Lu1—O42	72.73 (10)	C18—C17—C16	120.2 (5)
O41—Lu1—O22	144.98 (9)	C18—C17—H17A	119.9
O11—Lu1—O22	70.70 (9)	C16—C17—H17A	119.9
O21—Lu1—O22	76.01 (9)	C43B—O43—P4	120.7 (8)
O31—Lu1—O22	73.62 (9)	C43A—O43—P4	118.8 (5)
O32—Lu1—O22	72.03 (9)	C28—C27—C26	120.1 (5)
O12—Lu1—O22	129.42 (10)	C28—C27—H27A	119.9
O42—Lu1—O22	130.44 (9)	C26—C27—H27A	119.9
O43—C43A—H43A	109.5	O24—C24—H24A	109.5
O43—C43A—H43B	109.5	O24—C24—H24B	109.5
H43A—C43A—H43B	109.5	H24A—C24—H24B	109.5
O43—C43A—H43C	109.5	O24—C24—H24C	109.5
H43A—C43A—H43C	109.5	H24A—C24—H24C	109.5
H43B—C43A—H43C	109.5	H24B—C24—H24C	109.5
O43—C43B—H43D	109.5	C17—C18—C19	120.8 (5)
O43—C43B—H43E	109.5	C17—C18—H18A	119.6
H43D—C43B—H43E	109.5	C19—C18—H18A	119.6
O43—C43B—H43F	109.5	C21—C22—Cl23	112.0 (3)
H43D—C43B—H43F	109.5	C21—C22—Cl22	112.1 (3)
H43E—C43B—H43F	109.5	Cl23—C22—Cl22	109.2 (2)
O31—P3—O33	113.02 (17)	C21—C22—Cl21	107.0 (3)
O31—P3—O34	110.61 (16)	Cl23—C22—Cl21	107.9 (2)
O33—P3—O34	102.89 (18)	Cl22—C22—Cl21	108.4 (2)
O31—P3—N3	119.15 (16)	C36—C37—C38	120.0 (4)
O33—P3—N3	102.54 (17)	C36—C37—H37A	120.0
O34—P3—N3	107.14 (18)	C38—C37—H37A	120.0
C31—O32—Lu1	133.1 (3)	C41—C42—Cl3A	108.3 (2)
C41—O42—Lu1	135.7 (3)	C41—C42—Cl1B	117.2 (8)
C21—N2—P2	122.1 (3)	C41—C42—Cl3B	111.3 (6)
C11—O12—Lu1	135.1 (3)	Cl1B—C42—Cl3B	113.7 (9)
P1—O11—Lu1	134.02 (16)	C41—C42—Cl2B	110.4 (4)
P4—O41—Lu1	135.81 (16)	Cl1B—C42—Cl2B	105.3 (9)
C24—O24—P2	120.6 (3)	Cl3B—C42—Cl2B	96.7 (7)
C11—C12—Cl11	113.8 (3)	C41—C42—Cl1A	107.1 (2)
C11—C12—Cl12	110.3 (3)	Cl3A—C42—Cl1A	109.95 (17)
Cl11—C12—Cl12	108.8 (2)	C41—C42—Cl2A	113.0 (2)
C11—C12—Cl13	106.7 (3)	Cl3A—C42—Cl2A	110.40 (19)
Cl11—C12—Cl13	109.3 (2)	Cl1A—C42—Cl2A	108.00 (15)
Cl12—C12—Cl13	107.7 (3)	C40—C35—C36	120.1 (4)
C33—O33—P3	118.2 (3)	C40—C35—P5	121.5 (3)
C23—O23—P2	117.0 (3)	C36—C35—P5	118.3 (3)
C34—O34—P3	123.4 (3)	O12—C11—N1	132.8 (4)
C13—O13—P1	118.7 (3)	O12—C11—C12	113.3 (4)
C20—C19—C18	119.5 (5)	N1—C11—C12	113.9 (4)
C20—C19—H19A	120.2	O33—C33—H33A	109.5
C18—C19—H19A	120.2	O33—C33—H33B	109.5
C21—O22—Lu1	131.7 (2)	H33A—C33—H33B	109.5
O32—C31—N3	131.5 (4)	O33—C33—H33C	109.5
O32—C31—C32	114.0 (3)	H33A—C33—H33C	109.5
N3—C31—C32	114.4 (3)	H33B—C33—H33C	109.5
C35—C40—C39	119.3 (4)	C11—N1—P1	121.3 (3)
C35—C40—H40A	120.3	C41—N4—P4	120.0 (3)
C39—C40—H40A	120.3	C25—C26—C27	118.8 (4)
P3—O31—Lu1	133.10 (15)	C25—C26—H26A	120.6
C14—O14—P1	118.4 (3)	C27—C26—H26A	120.6
C16—C15—C20	120.5 (4)	C37—C36—C35	119.9 (4)
C16—C15—P5	121.6 (3)	C37—C36—H36A	120.0
C20—C15—P5	117.8 (3)	C35—C36—H36A	120.0
P2—O21—Lu1	133.92 (16)	C50—C45—C46	119.6 (4)
O22—C21—N2	132.0 (4)	C50—C45—P5	116.9 (3)
O22—C21—C22	115.7 (3)	C46—C45—P5	123.3 (3)
N2—C21—C22	112.3 (3)	C30—C25—C26	120.7 (4)
O13—C13—H13A	109.5	C30—C25—P5	119.1 (3)
O13—C13—H13B	109.5	C26—C25—P5	120.2 (3)
H13A—C13—H13B	109.5	C47—C46—C45	119.3 (4)
O13—C13—H13C	109.5	C47—C46—H46A	120.3
H13A—C13—H13C	109.5	C45—C46—H46A	120.3
H13B—C13—H13C	109.5	C29—C30—C25	119.9 (4)
C44—O44—P4	120.3 (3)	C29—C30—H30A	120.1
C15—C16—C17	119.1 (5)	C25—C30—H30A	120.1
C15—C16—H16A	120.5	C27—C28—C29	120.9 (4)
C17—C16—H16A	120.5	C27—C28—H28A	119.6
C19—C20—C15	119.8 (5)	C29—C28—H28A	119.6
C19—C20—H20A	120.1	C47—C48—C49	120.5 (4)
C15—C20—H20A	120.1	C47—C48—H48A	119.8
C39—C38—C37	120.0 (4)	C49—C48—H48A	119.8
C39—C38—H38A	120.0	C30—C29—C28	119.5 (4)
C37—C38—H38A	120.0	C30—C29—H29A	120.2
C48—C47—C46	120.5 (4)	C28—C29—H29A	120.2
C48—C47—H47A	119.8	O14—C14—H14A	109.5
C46—C47—H47A	119.8	O14—C14—H14B	109.5
C38—C39—C40	120.5 (4)	H14A—C14—H14B	109.5
C38—C39—H39A	119.7	O14—C14—H14C	109.5
C40—C39—H39A	119.7	H14A—C14—H14C	109.5
C31—C32—Cl32	113.9 (3)	H14B—C14—H14C	109.5
C31—C32—Cl33	110.9 (3)	C45—C50—C49	120.7 (4)
Cl32—C32—Cl33	107.4 (2)	C45—C50—H50A	119.6
C31—C32—Cl31	106.1 (3)	C49—C50—H50A	119.6
Cl32—C32—Cl31	108.5 (2)	C48—C49—C50	119.2 (4)
Cl33—C32—Cl31	109.9 (2)	C48—C49—H49A	120.4
C31—N3—P3	118.4 (3)	C50—C49—H49A	120.4
O42—C41—N4	132.5 (4)	O23—C23—H23A	109.5
O42—C41—C42	113.0 (3)	O23—C23—H23B	109.5
N4—C41—C42	114.4 (3)	H23A—C23—H23B	109.5
O34—C34—H34A	109.5	O23—C23—H23C	109.5
O34—C34—H34B	109.5	H23A—C23—H23C	109.5
H34A—C34—H34B	109.5	H23B—C23—H23C	109.5
O34—C34—H34C	109.5	O44—C44—H44A	109.5
H34A—C34—H34C	109.5	O44—C44—H44B	109.5
H34B—C34—H34C	109.5	H44A—C44—H44B	109.5
O41—P4—O43	112.25 (16)	O44—C44—H44C	109.5
O41—P4—O44	110.09 (17)	H44A—C44—H44C	109.5
O43—P4—O44	105.16 (18)	H44B—C44—H44C	109.5
O41—P4—N4	120.01 (17)		
			
O31—P3—O33—C33	61.0 (3)	O22—C21—C22—Cl23	21.9 (5)
O34—P3—O33—C33	−58.3 (3)	N2—C21—C22—Cl23	−160.4 (3)
N3—P3—O33—C33	−169.4 (3)	O22—C21—C22—Cl22	145.0 (3)
O31—P3—O34—C34	3.1 (5)	N2—C21—C22—Cl22	−37.2 (5)
O33—P3—O34—C34	124.1 (4)	O22—C21—C22—Cl21	−96.2 (4)
N3—P3—O34—C34	−128.2 (4)	N2—C21—C22—Cl21	81.5 (4)
Lu1—O32—C31—N3	31.3 (6)	C39—C38—C37—C36	1.6 (7)
Lu1—O32—C31—C32	−146.0 (3)	O42—C41—C42—Cl3A	−46.1 (3)
O33—P3—O31—Lu1	137.0 (2)	N4—C41—C42—Cl3A	136.8 (3)
O34—P3—O31—Lu1	−108.3 (2)	O42—C41—C42—Cl1B	112.4 (9)
N3—P3—O31—Lu1	16.5 (3)	N4—C41—C42—Cl1B	−64.6 (9)
Lu1—O22—C21—N2	−17.2 (7)	O42—C41—C42—Cl3B	−20.9 (7)
Lu1—O22—C21—C22	160.1 (3)	N4—C41—C42—Cl3B	162.0 (7)
P2—N2—C21—O22	−6.7 (7)	O42—C41—C42—Cl2B	−127.1 (6)
P2—N2—C21—C22	176.0 (3)	N4—C41—C42—Cl2B	55.9 (6)
C20—C15—C16—C17	−1.9 (7)	O42—C41—C42—Cl1A	72.4 (3)
P5—C15—C16—C17	175.4 (4)	N4—C41—C42—Cl1A	−104.6 (3)
C18—C19—C20—C15	1.1 (9)	O42—C41—C42—Cl2A	−168.8 (3)
C16—C15—C20—C19	0.8 (8)	N4—C41—C42—Cl2A	14.2 (4)
P5—C15—C20—C19	−176.6 (4)	C39—C40—C35—C36	2.9 (7)
C37—C38—C39—C40	−1.2 (8)	C39—C40—C35—P5	179.2 (4)
C35—C40—C39—C38	−1.0 (7)	C45—P5—C35—C40	114.3 (4)
O32—C31—C32—Cl32	−150.1 (3)	C25—P5—C35—C40	−5.9 (4)
N3—C31—C32—Cl32	32.1 (5)	C15—P5—C35—C40	−126.4 (4)
O32—C31—C32—Cl33	−28.8 (4)	C45—P5—C35—C36	−69.2 (4)
N3—C31—C32—Cl33	153.5 (3)	C25—P5—C35—C36	170.5 (3)
O32—C31—C32—Cl31	90.6 (4)	C15—P5—C35—C36	50.1 (4)
N3—C31—C32—Cl31	−87.2 (4)	Lu1—O12—C11—N1	−3.7 (7)
O32—C31—N3—P3	1.4 (6)	Lu1—O12—C11—C12	175.0 (3)
C32—C31—N3—P3	178.7 (3)	Cl11—C12—C11—O12	171.8 (3)
O31—P3—N3—C31	−24.1 (4)	Cl12—C12—C11—O12	49.2 (4)
O33—P3—N3—C31	−149.7 (3)	Cl13—C12—C11—O12	−67.6 (4)
O34—P3—N3—C31	102.3 (3)	Cl11—C12—C11—N1	−9.2 (5)
Lu1—O42—C41—N4	8.0 (7)	Cl12—C12—C11—N1	−131.8 (3)
Lu1—O42—C41—C42	−168.3 (2)	Cl13—C12—C11—N1	111.4 (4)
Lu1—O41—P4—O43	−120.8 (2)	O12—C11—N1—P1	7.2 (7)
Lu1—O41—P4—O44	122.4 (2)	C12—C11—N1—P1	−171.6 (3)
Lu1—O41—P4—N4	0.6 (3)	O11—P1—N1—C11	3.1 (4)
C44—O44—P4—O41	20.0 (5)	O14—P1—N1—C11	−124.5 (4)
C44—O44—P4—O43	−101.1 (4)	O13—P1—N1—C11	128.2 (4)
C44—O44—P4—N4	150.4 (4)	O42—C41—N4—P4	−0.9 (7)
Lu1—O21—P2—O24	135.4 (2)	C42—C41—N4—P4	175.4 (2)
Lu1—O21—P2—O23	−113.7 (2)	O41—P4—N4—C41	−3.3 (4)
Lu1—O21—P2—N2	10.7 (3)	O43—P4—N4—C41	122.5 (3)
C24—O24—P2—O21	−179.8 (4)	O44—P4—N4—C41	−127.6 (3)
C24—O24—P2—O23	62.3 (4)	C28—C27—C26—C25	−2.7 (8)
C24—O24—P2—N2	−49.8 (4)	C38—C37—C36—C35	0.2 (7)
C23—O23—P2—O21	64.1 (3)	C40—C35—C36—C37	−2.5 (7)
C23—O23—P2—O24	179.3 (3)	P5—C35—C36—C37	−179.0 (3)
C23—O23—P2—N2	−66.7 (3)	C25—P5—C45—C50	−41.1 (4)
C21—N2—P2—O21	10.3 (4)	C15—P5—C45—C50	79.3 (4)
C21—N2—P2—O24	−114.0 (3)	C35—P5—C45—C50	−163.6 (4)
C21—N2—P2—O23	137.3 (3)	C25—P5—C45—C46	142.4 (4)
Lu1—O11—P1—O14	109.5 (2)	C15—P5—C45—C46	−97.2 (4)
Lu1—O11—P1—O13	−137.5 (2)	C35—P5—C45—C46	19.8 (4)
Lu1—O11—P1—N1	−17.4 (3)	C27—C26—C25—C30	0.4 (7)
C14—O14—P1—O11	−50.8 (3)	C27—C26—C25—P5	−177.6 (4)
C14—O14—P1—O13	−170.8 (3)	C45—P5—C25—C30	−60.7 (4)
C14—O14—P1—N1	81.0 (3)	C15—P5—C25—C30	179.3 (3)
C13—O13—P1—O11	−42.9 (4)	C35—P5—C25—C30	61.5 (4)
C13—O13—P1—O14	75.5 (4)	C45—P5—C25—C26	117.4 (4)
C13—O13—P1—N1	−171.4 (4)	C15—P5—C25—C26	−2.6 (4)
C16—C15—P5—C45	−5.9 (4)	C35—P5—C25—C26	−120.4 (4)
C20—C15—P5—C45	171.5 (4)	C48—C47—C46—C45	0.1 (7)
C16—C15—P5—C25	112.9 (4)	C50—C45—C46—C47	2.4 (7)
C20—C15—P5—C25	−69.8 (4)	P5—C45—C46—C47	178.8 (3)
C16—C15—P5—C35	−126.0 (4)	C26—C25—C30—C29	2.2 (7)
C20—C15—P5—C35	51.4 (4)	P5—C25—C30—C29	−179.7 (4)
C15—C16—C17—C18	1.2 (8)	C26—C27—C28—C29	2.3 (8)
O41—P4—O43—C43B	−19 (3)	C46—C47—C48—C49	−3.5 (8)
O44—P4—O43—C43B	101 (3)	C25—C30—C29—C28	−2.6 (7)
N4—P4—O43—C43B	−150 (3)	C27—C28—C29—C30	0.3 (8)
O41—P4—O43—C43A	−56.0 (15)	C46—C45—C50—C49	−1.5 (8)
O44—P4—O43—C43A	63.6 (15)	P5—C45—C50—C49	−178.2 (4)
N4—P4—O43—C43A	173.3 (14)	C47—C48—C49—C50	4.4 (9)
C16—C17—C18—C19	0.7 (10)	C45—C50—C49—C48	−1.9 (9)
C20—C19—C18—C17	−1.9 (10)		

### Hydrogen-bond geometry (Å, º)

**Table:**

*D*—H···*A*	*D*—H	H···*A*	*D*···*A*	*D*—H···*A*
C26—H26*A*···O44	0.95	2.56	3.402 (6)	148
C14—H14*B*···O22	0.98	2.61	3.561 (5)	164
C43*B*—H43*E*···Cl12	0.98	2.93	3.73 (6)	140
C20—H20*A*···Cl1*A*	0.95	2.83	3.694 (5)	152
C34—H34*B*···O31	0.98	2.36	2.887 (6)	113
C19—H19*A*···O21	0.95	2.56	3.479 (6)	162
C43*B*—H43*D*···Cl32^i^	0.98	2.86	3.37 (2)	113
C40—H40*A*···Cl11^ii^	0.95	2.94	3.676 (4)	135
C23—H23*A*···Cl3*A*^iii^	0.98	2.99	3.768 (5)	137
C33—H33*A*···Cl33^iv^	0.98	2.89	3.597 (5)	130
C50—H50*A*···O23^v^	0.95	2.56	3.355 (5)	142
C49—H49*A*···N2^v^	0.95	2.71	3.540 (6)	146
C49—H49*A*···Cl21^v^	0.95	2.99	3.795 (5)	143
C17—H17*A*···O33^vi^	0.95	2.88	3.383 (6)	114
C17—H17*A*···N3^vi^	0.95	2.68	3.424 (6)	136

Symmetry codes: (i) −*x*+1, −*y*+1, −*z*+1; (ii) −*x*, *y*−1/2, −*z*+1/2; (iii) −*x*, −*y*+1, −*z*; (iv) −*x*+1, *y*+1/2, −*z*+1/2; (v) *x*, −*y*+1/2, *z*+1/2; (vi) −*x*+1, *y*−1/2, −*z*+1/2.
